# Factors that determine mental health professionals’ decision to support home-based video consultations – A qualitative study

**DOI:** 10.3389/fpsyt.2022.984026

**Published:** 2022-09-28

**Authors:** Anne Marie Moeller, Jens Peter Hansen, Pernille Tanggaard Andersen

**Affiliations:** ^1^Unit for Health Promotion Research, Department of Public Health, University of Southern Denmark, Odense, Denmark; ^2^Research Unit for Telepsychiatry and E-Mental Health, Centre for Telepsychiatry, The Mental Health Services in the Region of Southern Denmark, Odense, Denmark; ^3^Psychiatric Research Unit Esbjerg, The Mental Health Services in the Region of Southern Denmark, Esbjerg, Denmark; ^4^Department of Clinical Research, Center for Clinical Nursing Research, University of Southern Denmark, Odense, Denmark

**Keywords:** video consultation, mental health professionals, access, health care service, Grounded Theory

## Abstract

**Introduction:**

Using videoconferencing for consulting with patients in the mental health services has been shown in interventions to be similarly effective as when meeting in person. In practice, it often makes more sense to use video consultations with patients in a more flexible way than interventions permit. The aim of this study was to investigate what constitutes a professional video consultation from the perspectives of mental health professionals and explore what is of importance for the establishment and realization of video consultations in practice.

**Materials and methods:**

A Grounded Theory methodology approach based on Corbin and Strauss was used. Data collection consisted of participant observations of introductory events followed by individual interviews with mental health professionals who had used video consultations with patients.

**Findings:**

Mental health professionals believed that a professional video consultation was one that was not inferior to an in-person consultation but offered something else, such as more and easier access, accommodating patients’ needs and wishes. At the same time, it should not interfere with the treatment quality, e.g., by hampering communication and therapeutic tasks. The expected treatment quality was based on an individual assessment of the patient and varied from clinician to clinician. The implementation process and support which the organization provided affected the clinicians’ attitudes as well as the clinicians’ experiences and hence how the clinicians assessed the quality of the service.

**Conclusion:**

Perceived usefulness, patient demands, and close IT support will positively impact the establishment and realization of video consultations whereas high workload and technical problems would hamper it.

## Introduction

The use of home-based video consultations in the mental health services has several benefits. Aside from increasing access, as was the case during the COVID-19 restrictions (1, 2), it has proven to reduce waiting times and save costs for patients (3), and it has the potential to make treatments more person-centered if used to cater to patients’ individual needs. Moreover, evidence has established that the clinical effect of using home-based video consultations among different patient groups is similar to in-person consultations at clinics, satisfaction and acceptance are high, and the therapeutic alliance can be kept strong (4–8). Qualitative studies indicate that patients find video consultations acceptable when they experience barriers to in-person services and when it is perceived to not interfere with their treatment (9). Less is known about how to use video consultations in usual practice, the role of providers’ voluntariness of use on their acceptance, and how social influence from leadership and contextual factors impact providers’ attitudes (10). Further, a barrier to implementing video consultations into traditional mental health care services has been the mental health care professionals’ skeptical attitudes and perspectives. Given that clinicians function as gatekeepers for the use of video consultations (11), it is important to understand what their concerns are. At the same time, qualitative research exploring the perceptions, experiences, and satisfaction of clinicians in regard to video consultations is limited (12).

The aim of this study is to investigate what constitutes a professional video consultation with regard to its framework and content from the perspectives of mental health professionals. *Framework* refers to the organizational factors such as working procedures, support, and culture; and *content* refers to what is happening via the video consultation. It further explores what is of importance for the establishment and realization of video consultations to make it accepted among the clinicians. The investigation took place in a Danish setting and is based on an exploration of what considerations and experiences early adopters from a public mental health hospital had with video consultations used with adult outpatients. This will strengthen our understanding of the circumstances where video consultations will be considered meaningful and guide policymakers’ and managers’ decisions regarding initiatives to implement it in practice.

## Materials and methods

### Description of setting

In Denmark, most mental healthcare services are universal and publicly financed, based on the principles of free of charge and equal access for all citizens. Psychiatric hospitals are owned, managed, and financed by regions, which are governed by democratically elected councils. To access outpatient care, the patient needs a referral from selected professionals such as a general practitioner. The Region of Southern Denmark covers an area of 12,191 km^2^ with approx. 1.2 million people in 2021. The region has a large rural population (13), and it has been observed that distance to mental health clinics for outpatient care has a negative impact on the rates of visits, especially for people with lower socioeconomic status (14). To ensure easy access, the region has local psychiatric hospital centers in 13 cities across the region with specialized services in five geographically selected cities. It is a goal to treat as many as possible in outpatient care, since it is considered to be less intrusive and that it best supports the patient continuing their everyday lives (15). Further, the region has decided to include telepsychiatry in their mental health service strategy in order to improve accessibility and to strengthen the coherence in patients’ treatment courses. This resulted in the establishment of Centre for Telepsychiatry in 2013, facilitating the development and implementation of telepsychiatric solutions across the whole region (16).

Among other things, since 2015, clinicians have been given the opportunity to consult their patients via videoconference while the patients were at home, as a substitute or supplement to in-person consultations. These consultations could consist of psychiatric assessments, psychoeducation, medication management, psychosocial support, and psychotherapy. Hospital managers advocated strongly for the use of video consultations, but no specific demands were imposed on clinicians. Hospital managers made sure that video consultations were placed on an equal footing with in-person consultations regarding legislative demands and better in regard to reimbursement as an incentive for its use. Whole departments or teams in the departments who felt ready to implement the video consultation solution contacted a telepsychiatric implementation team who facilitated introduction, installation, education, and support of video consultations. Actual working procedures in that regard varied between each team and depended on team managers’ and department managers’ prioritization.

It was up to each clinician to decide if they wanted to offer their patients video consultations. There were no regulations or screening tools the therapists could use; it was an individual decision. When they deemed a patient eligible, they would introduce it. If the patient accepted, he or she received instructions on how to install the video program and perform troubleshooting, together with a personal username and password. The patients used their own personal devices compatible with video conferencing. The video consultations were scheduled, and only the clinicians were able to call the patients via video.

### Study design

A Grounded Theory methodology approach based on Corbin and Strauss was chosen (17). When using Grounded Theory, data are collected and interpreted in an inductive way. Theoretical sampling is used where collection and analysis of data are concurrent, and where the analysis is leading the further collection of data. Data are analyzed via open coding where they are put into parts, then with axial coding, where connections between codes are drawn, followed by selective coding, where the codes are used to form categories. Categories are developed based on their properties and dimensions, and sampling of incidents continues until the category is considered to be sufficiently developed. Important analytic strategies include making data comparisons and asking questions of the data. To construct theory, the context and process must be studied, among other things by coding conditions, action-interaction, and consequences around a category (17).

### Data collection and analysis

The first author did participant observations in two departments in the Mental Health Service in the Region of Southern Denmark where she had informal conversations with clinicians, managers, and the implementation team. She participated in workshops, introduction to video consultations at the clinics, technical start-up meetings, team meetings where video consultations were on the agenda, and offered videoconference support to the clinicians. Further, seven individual interviews were made with hospital, department, and team managers, and telepsychiatric facilitators to get an understanding of the background and visions behind video consultations. The field observation took place during a period of 18 months covering parts of 2016–2017. Field notes were written down after each meeting.

Inspired by the constant comparative method, during and after each field observation, incidents were noted down and assembled into temporary categories. The incidents were then compared back and forth with previously incidents to answer the question “what is happening here?” and used to refine the categories. Categories were discussed between first and third author and preliminary analysis were done. Two initial categories were defined and included “professional attitudes” where varying degrees of both skeptical and pragmatic attitudes were uncovered, and “assessed treatment quality” where different types of patients were considered more or less suitable, where the therapeutic relationship was expected to be more or less affected, and whether the modality were considered safe for the patients.

An example of how the categories were derived from the fieldwork:

*“Together with an implementation consultant, I approached different teams in a department to help install the video program and video equipment on the clinicians’ computers in their offices. In one office, a clinician, who was mainly working with patients with a schizophrenic disorder, stated that we were allowed to install the program on her computer, but she had no intention of using it due to the type of patients she had. She pointed toward the smoke detector in the ceiling, a dark grey round box with a small red flashing light, and said that some of her patients thought they were being recorded through that, and she, therefore, thought that it would be of no good for her patients to talk through a screen”.*

*Memo: The clinician is skeptical about using video consultations. She worries about what is right for her patients and whether video consultation could do harm. I consider it professional to be skeptical about trying out new things in order to protect her patients. I also wonder how the clinicians are supposed to choose their patients. It appears that managers still need to communicate what the purpose with video consultations is since it is not clear for this clinician.*

*Temporary category: Professional skepticism. Concept: Withholding video consultation option.*

Initially, the focus was on the clinicians’ experiences with the use of video consultations. During the field observations it became clear that the therapists were concerned about how they were supposed to select patients. Using the principle of theoretical sampling, this narrowed the focus, and the following data collection was thus guided by the new knowledge.

To further explore how the clinicians selected their patients and how they perceived the quality of the video consultations they had conducted, clinicians who already had done a video consultation with at least one patient in the patient’s own home were invited to participate in individual interviews. Some clinicians had used videoconferencing with hospitalized patients and their career for specialist support, but the interviews focused on their experiences with patients seen in their own home. Outpatients were only able to access their treatment either in person at the clinic or via a videoconference received at the patient’s own device. Various types of clinicians were included to explore variations and similarities across occupational background and teams. A semi-structured interview guide was developed, based on the questions the preliminary analysis gave rise to. Topics in the guide included: (1) What do you think about video consultations? (2) How was it introduced to you? (3) How do you choose to whom you offer video consultations? And (4) How did you experience communicating with the patient via video and how did that affect the therapeutic relationship? Interviews were conducted at each clinician’s office and lasted around 1 h. After each interview, initial concepts were noted down and used to direct specific focus in the coming interviews. All interviews were recorded and transcribed. After all interviews were collected, data from each interview were analyzed to refine the established categories (17). Examples of coding can be seen in Table 1.

**TABLE 1:** Examples of coding.

Quote	Code	Category
*I: “And then I had one with a personality disorder, eh, and that was difficult because sometimes there was substance abuse involved, but it was good in relation to that*… *if she didn’t get these [video] conversations, then she stayed away [from treatment]”*	Using video consultations reduces no-shows for some patients, and a video consultation is better than a no-show	The clinician’s total assessment of the patient’s problems affects the expected treatment quality when using video
*A: “Yes, you told me about the patients you chose, you said that your relationship was good before you started.”* *I: “Yes, and that’s more because I find that it’s in order to be calm about it, because IT is tricky sometimes, and there are sometimes you don’t call, and sometimes their internet connection is inferior, and if you don’t know the patient well enough to know, ok, do I sit with one who is suicidal and suddenly feel pressured in it, and can’t get in contact with me, or is it someone who totally panics that ‘it is me’ (*…*)”*	Knowing the patient increases the confidence in making a risk assessment of how the patient will tolerate a disconnection	The clinician’s total assessment of the patient’s problems affects the expected treatment quality when using video

The first author conducted all the interviews. She has a background in science of public health and has, therefore, no experience with consultations of mental health patients. This gave her an outsider perspective, which perhaps created a more objective look at practice but also made her naïve to informal cultural practice and jargon. The outsider perspective may have changed over time. She was employed within the organization and was seen by the participants as a colleague from a different department. This may have increased the participants’ trust in the interviewer.

### Participants

15 clinicians were invited via e-mail to participate in the individual interviews. 11 responded to the request and consented. In the final interviews, no new substantial concepts in relation to the research question emerged, and no more clinicians were contacted. Participant characteristics can be seen in Table 2.

**TABLE 2:** Participants in formal individual interviews.

**Gender**		
	Female	9
	Male	2

**Occupation**		

	Psychiatric nurse	5
	Psychiatrist	2
	Psychologist	1
	Psychotherapist	1
	Occupational therapist	1
	Medical social worker	1

**Number of patients seen *via* a videoconference**		

	Mean	≈3
	Range	1–6

**Patients’ diagnoses of those seen *via* videoconference**		

	Anxiety disorders including obsessive-compulsive disorder, depression, bipolar disorder, schizophrenia, eating disorders, Asperger’s disorder, and personality disorders	

**Services given *via* videoconference**		

	Medication adjustment, psychoeducation, cognitive therapy, follow-up sessions, preventive conversations, and social support	

**How video consultations are used**		

	In a combination between video and physical presence Start-up courses or final courses The whole treatment course	

**What video consultations are used for**		

	Replaces consultations with physical attendance Adds additional consultations Used instead of a cancelation	

### Ethical considerations

The Regional Committee of Health Research Ethics for Southern Denmark was queried for ethical approval (no. S-20162000-29) and the project was reported to the Danish Data Protection Agency. Department managers were notified about the project, and team managers and local research coordinators approved of the study. During field work, the researcher who did the data collection presented herself as a researcher. Participants in interviews were informed in writing of the purpose of the study in their invitation to participate and orally before the interviews. They were assured anonymity, voluntary participation, and the right to withdraw their consent. They all gave written informed consent before the interviews began.

## Findings

### Clinicians’ patient screening process

**FIGURE 1:**
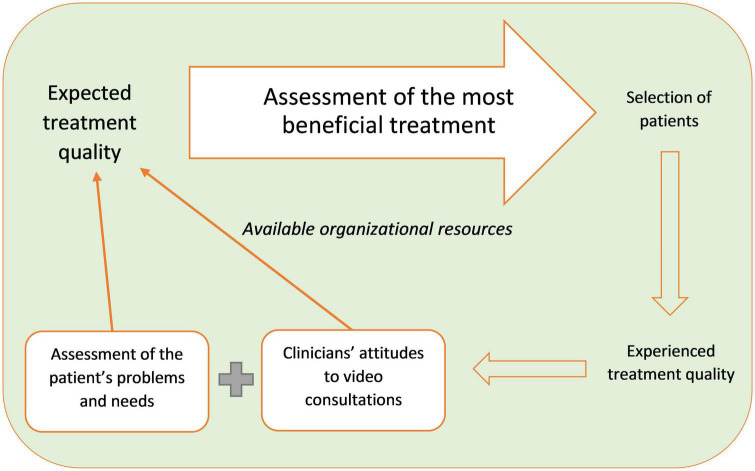
Clinicians’ patient screening process.

From the analysis, the core category, “assessment of the most beneficial treatment,” was generated. It derived from the following categories: “expected treatment quality,” “assessment of the patient’s problems and needs,” “clinicians’ attitudes to video consultations,” “available organizational resources,” and “experienced treatment quality.” The categories are connected as follows: When the clinicians decided whether they wanted to offer a video consultation to a patient, they assessed how they expected this would affect the treatment quality of this particular patient. The assessment was based on the patient’s problems and needs. They balanced barriers and advantages, and when the total quality was expected to be better than without it, they offered it. It can be complex to counterbalance barriers with advantages; hence, it varied from clinician to clinician. Variations were based on the clinicians’ different attitudes toward video consultations, which were both guided by individual qualities, such as preferences and technical skills, and organizational conditions, such as the way video consultations were implemented in the system. The available organizational resources, such as support, working conditions and the videoconferencing system, restricted the scope of what was feasible. As the clinicians acquired experience with using video consultations, their attitudes became more positive or negative with regard to what video consultations were able to accomplish. Figure 1 illustrates the process that the clinicians go through when assessing whether they want to offer a video consultation to a patient.

### Expected treatment quality

The clinicians assessed that the use of video consultation could improve the treatment quality when the patient experienced that access to their treatment became easier and more readily available. With increased accessibility, the clinicians expected the patients to be more satisfied and that there would be fewer no-shows. This could safeguard continuity in the treatment course, which would strengthen the therapeutic process. An example:

*A: Do you also use it to have more frequent contact?*

*I: Yes, with some of them, so you can follow up on them more often, yes. And I think you can say it’s also that frame of reference that we sort of work within, you may say, when you mainly work cognitively, then preferably not more than a month should pass between [sessions], or else there’s just too much, then there will be too much spam in the intervening period, so, the more often you actually can have a conversation, the better, then you can speed up the course a bit and help them (*…*).* Participant 5, psychiatric nurse.

In addition, video consultations could be offered as extra consultations since they are less resource-demanding and hence serve as an expansion of the existing offering. Conversely, the clinicians found the quality deteriorated when video contact prevented or hampered significant treatment components and when the technology was unstable, since access to the consultation would be reduced and the offering became unreliable.

### Assessment of the patient’s problems and needs

The clinicians’ point of departure when considering using video consultations was that the patient experienced a problem that complicated or prevented physical attendance at the clinic. This was both related to the lack of specific transportation options, expenses, and the time spent on the transport; mental problems patients might experience during transportation or their stay at the clinic; and social and cognitive difficulties with remembering the appointment and organizing transportation. This was the main reason why the clinicians considered offering video consultations. An example:

*I: But I can easily imagine that after the first personal [in-person] conversation that I continue the diagnosing [over video], but that would require that the patient said: ‘I find it difficult to turn up’, then I would order a [video] license. If the patient doesn’t mention anything, then they’ll just receive their next appointment to turn up here.* Participant 6, psychiatrist.

In this case, the psychiatrist only offered a video consultation to patients who expressed an obstacle to access the clinic. Given that the psychiatrist believed that he could make a better assessment of the patient in person, he did not suggest it proactive to patients who might from their perspectives have benefited from its use. Subsequently, the clinicians assessed how they thought the video consultations would affect the patients’ course of treatment, based on the patients’ mental condition and social competencies. For example, suicidal patients, psychotic patients, and patients with a substance abuse problem were mentioned as among those patients the clinicians deemed unsuited for video consultations for fear that they would be unable to observe the patient sufficiently and hence unable to intervene if necessary. Another example included patients with social phobia. When the patients could not leave their home, video consultations could be used at the beginning of the course of treatment, and then they could practice showing up at the clinic in due course. Furthermore, the patients with social phobia were more relaxed at home, which made it easier for them to work with some issues. These elements suggested that video consultations could improve the quality of treatment. An example of where the quality was restricted included patients with a major depression. They are typically affected socially and tend to isolate themselves. In these cases, the clinicians typically considered it important that the patient show up at the clinic to maintain certain functional abilities. That is, when the patient has poor social capability, it is not considered beneficial for the patient to meet over a video link, unless it is the only option for therapy. Some clinicians chose for that reason to first consider video consultations after social competencies have been practiced. Of other social competencies, the way the patients communicate also impacted on how the video consultations were assessed as beneficial. For example, a clinician mentioned that she did not want to offer video consultations to those of her patients who were very taciturn since the communication with that patient would be more supported by non-verbal language, which is less available via video. To some degree, it varied from clinician to clinician how they assessed the beneficial effect that meeting in person has on the patient’s social competencies and the need for exposure.

The patient’s interest in video consultations was also decisive for whether a video consultation was established. Most of the patients who were offered a video consultation wanted to make use of it, presumably due to the clinicians’ careful selection of patients they offered video consultations to. Some patients requested video consultations, which the clinicians in general were willing to meet to secure the patients’ engagement in their treatment and make them feel that their needs were met. In several cases, this was the reason why the clinician started to use video consultations with a patient. The clinicians were in general attentive to the patients’ requests, such as here:

*A: Of those you told about video, did anyone say that they didn’t want to?*

*I: No, not at all. These people, they’re very positive about it*… *especially when there’s a long journey to get here and it’s difficult*… *(*…*) so I have several [patients] who write: “well couldn’t we, could we possibly meet up on video today?” because some transport scheme went pear-shaped for them. And then we do it [video consultation]. So, it’s always at the patient’s request, if we end up using video.* Participant 7, Psychotherapist.

The clinicians’ experience with what affected the patients’ interest was, among other things, the patients’ IT skills. Especially younger patients under the age of 40 who are used to IT did not encounter any challenges with the technology. For patients who were less IT-savvy, the combination between IT challenges and the cognitive challenges their mental disorder may cause could be the reason why they were unable to cope with video consultations. However, the patients could bring their devices and get help from the clinician, or they had relatives who could help with the technology. A limitation was, though, that some patients did not have the right IT equipment and hence were excluded. These were often patients with severe disorders who were unable to work and hence had a low socioeconomic status. Some of these patients received home visits, and they may not be interested in changing those encounters to video. Nevertheless, some clinicians were able to offer consultations in addition to the usual care, which they would not have had the resources to do if they had to make a house call.

In summary, the clinicians assessed the patient’s total situation before they decided whether to offer a video consultation. They considered problems regarding transport, mental issues, personality, and interest, and how these things would affect the treatment quality.

### Clinicians’ attitudes to video consultations

The clinicians’ individual attitudes toward video consultations also affected how they assessed the treatment quality. Their attitudes were affected both by their individual competences (such as IT skills as well as their knowledge about and experience with video consultations) and the manner in which they were introduced to them.

When the clinicians were introduced to video consultations, many of them were initially skeptical and saw it as a threat to their patients, their integrity, and their working environment. At joint meetings, they expressed their frustrations and asked many questions about whether it really was the best option for their patients, and whether they as clinicians would be able to figure out the technical parts. This was also expressed in the interviews:

*A: And what did you think about it at that time [when she was introduced to video consultations]?*

*I: Ahh*… *I thought it was confusing and, ehm, I thought a lot about that with the relationships, how will it be when you do it over the computer, that is, how will your relationship be with the patients that you see on video? And then I was very scared about the technical stuff because I’m not very technology savvy, ha ha, when it comes to computers and plugs and headsets and webcam and stuff like that, so that, I was a bit, well actually scared of too. So, I think I was a bit skeptical, actually.* Participant 3, psychiatric nurse.

This participant was initially skeptical about how video consultations would affect her patients’ treatment. She also feared that she could not handle the technical part of the videoconferencing system which reinforced her skepticism. Many clinicians mentioned that they did not have good experience with the available IT support in other regards, and this might have intensified the fear of not being able to handle it. Other clinicians had a more pragmatic approach and expressed that video consultations gave them various options. An example:

*I: (*…*) so I feel sort of, I take it very from above and down, yes, when something new is coming. So, I don’t think: ‘oh no, something new is coming’. I think that it’s a way to keep up with reality of what’s happening IT-wise and what opportunities we have today.* Participant 1, medical social worker.

Whether the clinicians were skeptical or pragmatic influenced their decision to offer video consultations. Of those who were skeptical, some chose not to offer video consultations to anyone, while others picked out patients under specific conditions, e.g., if the patient had wanted the video option, or where video was the most clear-cut solution to a transportation issue. Those with a more pragmatic attitude offered video consultations to all with transportation difficulties, but also to patients who had several cancelations or no-shows, and they saw possibilities in offering additional video consultations consisting of follow-up conversations. The correlation is depicted in Table 3.

**TABLE 3:** The correlation between attitude and screening of patients to a video consultation.

Attitude	Assessed treatment quality	Selection of patients
Reserved	Poor	None
Skeptical	Poor or acceptable	A few
Pragmatic	Acceptable or good	Broad

The clinicians’ attitudes were, among other things, influenced by the workload they experienced before the video consultations were introduced and their coping strategies. Some therapists felt that they did not have the mental capacity to acquaint themselves with something new, even though it could potentially save time in the end. By turning down new things, they avoided additional workloads. Among the interviewees, those who defined themselves as having good IT skills felt less insecure using video consultations since they could acquaint themselves with the program and solve any difficulties faster, so they were less skeptical. However, more than half of the clinicians who already had tried video consultations described in the interviews that they did not have good IT skills. They often had more difficulties getting IT support when technical difficulties arose. Further, when the clinicians heard from colleagues that something did not work, they were less inclined to try it out themselves. They did not find it professional to offer something to the patients that would turn out to not work, and hence they stopped offering video consultations. They felt that they appeared unreliable, and they feared how that would affect the patients’ trust in their therapist and their course of treatment, which is important for the treatment effect.

### Available organizational resources

The available organizational resources (such as support, working conditions and the videoconferencing system) set the framework for what was possible, which influenced the clinicians’ attitudes and affected their practical experiences with video consultations. At the mental health hospitals, the clinicians had, in general, great autonomy over their consultations, and for that reason, the management’s enthusiasm had little impact on the clinicians’ attitudes. However, the management’s level of support had a major impact on how well the video consultations were implemented. It affected the clinicians’ trust in whether video consultations were possible, and that the treatment quality would remain the same, and in their willingness to change their existing practice. For example, the hospital management decided that video consultations should be reimbursed better than in-person consultations. Since phone calls did not receive any reimbursement, this incentive structure specifically made some clinicians consider whether they should convert some phone calls to video calls, still in consideration of the treatment quality. In that way, they could increase their productivity without increasing their effort because they were making the call regardless. An example:

*I: (*…*) It’s also a performance positioned system we are in, right, so you can say that many of the phone consultations you have, you can redefine to actual performance output [when using a video consultation]. I find that it’s a bit of a shame that it has to be like that, but it’s a fact.* Participant 5, psychiatric nurse.

Despite some incentive structures, video consultations were implemented in an experimental way where no guidelines were defined, and each clinician had to decide on their own what potentials they believed that video consultations could have, without being aware of the existing evidence in the field. Because of that, it has been challenging for the clinicians to know what to be guided by, which may have caused them to be more restrictive in their selection. The insecurity was, among other things, expressed as follows:

*I: The problem here is that you may not be that pressured, because when we got Cosmic [electronic patient journal], we had no other option but to use it. With Jabber [the video program], if you’re scared of it, you don’t mention it as an option to your patients. It is better if you show up in person, and I prefer that. So, maybe it isn’t used as much as it could and what seems sound, due to anxiety and how you deal with that.* Participant 6, psychiatrist.

At the same time, the clinicians worked under a service system where the throughput to a great extent was measured on the number of patient consultations. For that reason, the clinicians considered the patient consultations as productive, whereas training and problem solving in regard to the video program were considered counter-productive. Patient consultations can take place without video, and hence video consultations are not essential for the clinicians’ productivity. To the extent that the clinicians experienced that the training and problem solving took time away from patient consultations, they did not prioritize it unless they deemed it particularly beneficial to the patient that the conversation was over video. An example:

*I: (*…*) But then there has really been a long period where I didn’t use it because it’s so frustrating in a busy weekday that you actually have to spend even more time sometimes on such a [video] conversation than you have to on a regular conversation where people arrive in here due to that the technical stuff doesn’t work (*…*).* Participant 9, psychiatric nurse.

It has therefore been decisive for the use of video consultations that sufficient IT support was available and that the video program worked. The amount of IT support available was an important factor in how secure the clinicians felt when offering video consultations. When they knew who to contact to resolve technical issues, they need not feel nervous about how well the technology would work or whether they would have to spend time on their own troubleshooting issues. In general, it was important that the IT support was available immediately, since technical difficulties often occurred during an appointment with a patient. When the problems were not solved immediately, the clinicians had to change the appointment to a phone call. When the clinicians experienced difficulties, they often used the IT support physically closest to them, such as a clinical colleague. Some teams appointed superusers whose responsibility it was to help others with technical difficulties, and that worked well:

*I: And it’s, at any rate, nice as a therapist that I don’t have to struggle a lot with something technical, and there is a sort of service about it, and I can approach the conversation as any other conversation. Instead of fetching the patient in the waiting room, well then I just need to open my computer and open Jabber [the video program] (*…*).* Participant 8, psychologist.

Others had to find out on their own how to get help, which delayed the time span before a solution was found, during which they could not use video consultations:

*I: Well, I have some [patients] where we simply couldn’t get the tech working, and without finding out how that is interrelated. Well, that’s what I sometimes think, that we, as nurses, we’re left with this, to find out about all this technical stuff. (*…*) It takes time, and if you do call and can’t get through*… *or the patient’s equipment doesn’t work, or*… *there it becomes a time-waster, and we finally had to give up.* Participant 11, psychiatric nurse.

Reliable performance of the videoconferencing program, close IT support, and incentive structures all affected the clinicians’ attitudes toward video consultations and the experiences gained.

### Experienced treatment quality

The experience the clinicians gained with using video consultations has either strengthened or changed their attitudes toward video consultations and how they assessed how the video format would affect the treatment quality. This was both in regard to what type of patients they would use it with and what type of conversations they wanted to use it for. A clinician who was asked which advice she would give to those of her colleagues who had not used video yet, replied:

*I: Well, that you don’t have to be so skeptical, ha ha, as I was; I think, that is, that it actually works well, ehm, and that it’s a good supplement to them coming here; that is, to outpatient conversations, it’s a really good supplement, and the patients are happy about it. At least those I’ve had; they’ve been happy that there has been this offering (*…*).* Participant 3, psychiatric nurse.

Experience not only improved the clinicians’ technical skills so that IT difficulties took up less time; it also improved their way of communicating and interacting with the patients via video. In general, the clinicians had good experiences with using video consultations when all of the technical aspects worked and claimed that video consultations held a bigger potential than before they tried it.

Some of the experiences that the clinicians gained was that they often felt that the video consultations were more formal than in-person consultations. This was, among other things, because the clinician and the patient typically would sit straight in front of the screen where they would be able to see a passport photo segment of each other. In addition, they would typically use less small talk, which could shorten the conversations. Most often, the clinicians who did psychotherapy had made an agenda that both they and the patients would follow closely, and for that reason, they felt that the video conversations were more focused and effective compared to in-person consultations. The clinicians also felt that they did not get to know the patient as well. Small talk and how the clinicians acted in front of the screen could both be changed and tried out in new ways until they found an effective style. A clinician who had seen several patients via video explained that over time, she experienced that the video consultations in regard to content and timewise became more like the consultations she had in person.

The clinicians also experienced that some of the communication was attenuated and had to be replaced verbally. It put more of the responsibility for the communication on the patients, since it would be easier to disguise emotions. This made the clinicians worry whether they would be able to observe enough to interact most considerately in regard to the treatment. For that reason, they nearly always chose to use video consultations with patients with whom they already had established a relationship, allowing them to feel able to read the patient correctly and ensure a safe treatment. Two of the clinicians had tried a video course with one patient each where they did not meet in-person first. They both felt that they did not get to know their patient as well as their other patients, which made them feel insecure and led to a decision of not doing it again. Both clinicians experienced that their patients seemed pleased, that they were equally candid as their other patients, and that the patients improved so much that they were discharged from the hospital. The clinicians’ insecurity is thus not due to specific inexpedient events but rather because they felt less in charge of the treatment. Another clinician saw a potential in giving the patients more control and chose actively to offer video consultations to some of her patients who were gamers and hence used to using video for conversation. She told them that she was less IT-savvy than them and that she thought she could ask them for help in the event of any technical issues, effectively shifting the balance of power in their relationship. She believed the patients would feel that they were creating value for her in that it was not just them who needed help. Video consultations further allowed new opportunities since it sometimes was possible to get more information about the patients in their home. While some patients chose to display their home via the webcam, most of the time they would sit in front of the screen.

Several clinicians found that it was difficult to use the slate in therapy over video. They used the slate to draw and visualize cognitive models to explain how the cognitive therapy worked and how it applied to that individual’s problems. Instead, they chose to be more verbal and visualize cognitive models less. One therapist explained that it made the sessions shorter since they spent less time reflecting over concepts than they would if they looked at the visualized models on the slate. For that reason, several therapists decided to use video consultations for sessions with topics that had already been introduced. Then they could refer to models the patient was already familiar with:

*I: Sometimes I use the slate a lot, with models and stuff like that, and then I find it challenging because so far, we can’t run the video consultation while sharing our screen, (*…*) So thus, the level, also the quality of it, is not the same as if they were sitting inside the office. So, therefore, I must also choose: Should it be a consultation that’s a supporting conversation where you listen to how things have been since the last time, and how can you kind of work with them without there being too many slate projects?* Participant 2, psychiatric nurse.

In conclusion, the clinicians’ experiences showed that using videoconferencing with patients is a different way of communicating together. Having gained experience, the clinicians were able to adapt their sessions to the new modality and became more knowledgeable of how video consultations can be used in a meaningful way.

## Discussion

### Purpose and summary of key findings

During the COVID-19 crisis, many therapists have been compelled to cancel or virtualize sessions with patients. Concurrently with the relaxation of COVID-19 restrictions, psychiatric hospitals or community mental health centers, may want to continue some of these services to increase accessibility, and it is therefore relevant to look at which factors need to be available for it to be successful. In this study, factors that mental health professionals believed constitute a professional video consultation were investigated. It further explored what is of importance for the establishment and completion of video consultations in practice. The present study found that mental health professionals believed that a professional video consultation should not be inferior to an in-person consultation but should offer something else, such as more or easier access to the clinician. How the treatment quality is assessed to change over video varied between clinicians, and was, among other things, dependent on their attitudes, which again were shaped by the implementation process and the experiences they gained. Technical challenges and available IT support also played an important role in terms of deciding on whether or not to use video consultations.

### Significance of findings and integration with prior research

Our overall findings are in alignment with previous studies of mental health providers’ attitudes toward video consultations. Similarly, the studies found that clinicians believed video consultations could be effective and useful, since it facilitated access and met patient demands, but also that the medium was more impersonal and made it more difficult to detect non-verbal cues (10, 18). In general, the studies found that the overall attitudes were largely positive; providers were satisfied and favored the use of videoconferencing; and furthermore, the studies reported that the clinicians found it easy to use (10). In our study, participants encountered several technical challenges, and even though many found videoconferencing useful, they did not use it due to a combination of technological difficulties, having a stressful workday and little technical support. This highlights, as in other studies, how important facilitating factors are, such as IT support and training (10, 19). In addition, as found by others (20), we discovered that the clinicians who were initially skeptical but for some reason tried it out, were positively surprised that it was possible to sustain the quality of treatment via video. It therefore seems that the biggest challenge for implementation, besides sufficient access to technological support, is the lack of a clear purpose and knowledge of the benefits, which education and training could provide. Given the Danish infrastructure (where the distances from patients to the clinics are shorter than in many of the typically studied areas, such as rural Australia and USA), clinicians in Denmark may find that accessibility issues are less important, and their willingness to use video consultations may be more fragile. However, during the COVID-19 restrictions, video consultations have proven to be needed and an important part of disaster preparedness where patients safely could access health care services. Many therapists willingly converted to online modalities (21), and with this experience they might have more positive attitudes toward video consultations.

In this study, the providers voluntarily decided to whom they offered video consultations, and little has previously been studied in this context (10). We found that the clinicians have a strong professional identity and are reluctant to try something out if they do not see the immediate benefit compared to usual practice. This underpins the importance of sufficient education of the clinicians so they will have a realistic idea of the potential benefits. This study also contributes to new knowledge of the social influence of leadership in this context. The leaderships’ engagement and attempt at creating a positive attitude toward video consultations was somewhat unsuccessful. It might be due to that the implementation of video consultations was based on a political decision, making it a top-down controlled implementation, with the possibility that the management level closer to the clinicians did not fully commit to it. However, it did seem clear that the clinicians — due to the fact that it was voluntary — mostly were affected by the expected treatment outcome, which is in line with their professional identities. Many doubted on the effects and had a limited amount of time to acquaint themselves with this new procedure, which resulted in many clinicians who tried to get around it. This meant that their assessment of what was the best treatment were to a large degree shaped by their own insecurities and fear of not knowing how to use the technology as well as feeling a high workload. In the present study, the organization lacked infrastructure regarding the videoconferencing setup, which may have led to it being implemented in various ways. It was clear that the support needed to be close and immediate, or else it took time from patients, and hence it would negatively impact the overall treatment quality of the patients. What can be learned from this case is that implementers have to carefully explain why video consultations should be used and how that would affect the treatment quality. As clinicians are trained in evidence-based decision making, it would be beneficial to refer to the existing evidence and make clinical guidelines available and offer training. It is also important with investigative support, as many found it hard to spend time on solving technical issues and consequently threw in the towel. As found by others who investigated remote consultation services, for it to be successful there had to be staff members who could champion and support the innovation, such as a superuser (22), which is in alignment with our findings.

Another factor to be attentive to when considering offering video consultations to patients is that there needs to be a balance between the patient’s need and the clinicians’ assessment, which must rely on a mutual acceptance. However, there is the risk of one part being trumped. It can be difficult to assess the patient’s need, and they may disagree. In some cases, in this study, the patients exerted pressure on the clinicians to get a video session. Even though the clinicians had to be attentive to whether using video consultations would have any negative effect on the patients’ treatment course, the hospital managers had repeated that video consultations is something the hospital offer to all patients. For that reason, the clinicians felt that they had to offer it when a patient requested it. In most clinics there were pamphlets in the waiting rooms describing that it would be possible to get a video consultation. Therefore, their requests were always met unless technical issues prohibited it. However, therapists found that they needed to establish a relationship in person; nevertheless, they acknowledged that this was their personal need and they were uncertain of the patients’ needs, since those who only met via video seemed satisfied and at ease with the format. However, patients often express that they, too, prefer knowing the therapist before having a video consultation (9), and clinicians in other fields also prefer knowing their patients before switching to video (22). The relationship or therapeutic alliance is found to be determining for the clinical outcome (23), yet the clinical effect has been shown to be similar among consultations with physical presence and video consultations, but sometimes the alliance was better with physical presence than via video conferencing (5). Even though the alliance remained strong, it highlights that other things also affect the clinical outcome — such as convenience, easier access, and meeting patients’ individual needs and wishes.

Finally, consultations via videoconference may potentially facilitate access to mental health services; however, this does not apply for everyone. Home-based consultations require the right equipment and digital literacy, which is not always present among people with low socioeconomic status, socially marginalized people, ethnic minority groups, and the elderly (19, 24). Within the current system, we found that some patients did not have sufficient technology available. However, when the videoconferencing system that were used became compatible with smartphone this was solved.

### Strength and limitations

Participants were early adopters and the clinicians who opposed the most did not participate in the interviews. It was apparent, though, that several of the interviewees were skeptical and much less accepting of the modality than others. Understanding what made them adopt teletherapy might help understand the personal clinician barrier that needs to be overcome. Another limitation is that the clinicians had little experience with video consultations in general and hence were unable to take the full impact on the treatment and the relationship with their patients into consideration. The present study only focused on video consultations with patients in outpatient psychiatric hospital settings. The findings may therefore not be generalized to social psychiatry, and neither to support nor training between clinicians. However, we did include a range of professionals dealing with different types of treatment, from different teams as well, including treatment of a variety of patient categories. We did not find any diagnoses where video consultations would be of particular relevance or of no use. To understand when video consultations are meaningful for the individual, the study could be further strengthened by triangulation of the findings with other stakeholders in the mental health services, including patients and healthcare administrators using interviews, focus groups, and surveys.

## Conclusion

Our findings show that when clinicians are asked to voluntarily adopt video consultations with their patients, their decision to offer the service is based on an assessment of the most beneficial treatment for the individual patient. Both the patients’ problems and characteristics, as well as the clinicians’ attitudes, have an impact on the assessment. The clinicians’ attitudes are affected by the implementation process. In a system with high workload, the need to invest in extra training may outweigh the benefits of improved service delivery and will in that case negatively affect the clinicians’ attitudes toward the introduction of a new practice. The perceived usefulness and patient demands combined with close IT support will positively affect the clinicians’ attitudes. Overall, the clinicians had positive experiences with consulting the patients via video and maintaining a good working relationship with them. Consequently, the clinicians became more positive toward video consultations after having tried it, although technical challenges interfered with the process. Future research should further examine what education and training are needed and the effect on clinicians’ attitudes, and further investigate how video consultations can be used meaningfully, differentiated between patient groups.

## Data availability statement

The datasets presented in this article are not readily available because of ethical and privacy restrictions. Requests to access the datasets should be directed to the corresponding author.

## Ethics statement

Ethical review and approval was not required for the study on human participants in accordance with the local legislation and institutional requirements. The patients/participants provided their written informed consent to participate in this study.

## Author contributions

AM: conceptualization, investigation, data curation, formal analysis, and writing—original draft. JH and PA: formal analysis, supervision, and writing—review and editing. All authors contributed to the article and approved the submitted version.
